# Unexpected variations in translation initiation machinery

**DOI:** 10.1186/1471-2105-13-S18-A5

**Published:** 2012-12-14

**Authors:** Kyungtaek Lim, Yoshikazu Furuta, Ichizo Kobayashi

**Affiliations:** 1 Department of Medical Genome Sciences, Graduate School of Frontier Sciences, University of Tokyo, Minato-ku, Tokyo, 108-8639, Japan; 2 Institute of Medical Science, University of Tokyo, Minato-ku, Tokyo, 108-8639, Japan; 3 Department of Biophysics and Biochemistry, Graduate School of Science, University of Tokyo, Minato-ku, Tokyo, 108-8639, Japan

## Background

In prokaryotes, the Shine-Dalgarno (SD) sequence in the 5’ untranslated region (UTR) of mRNA forms a duplex with the anti-Shine-Dalgarno (anti-SD) sequence to initiate translation [1]. The mechanism has been regarded as universal, as the core of the anti-SD sequence (5’-CCTCC-3’, anti-SD motif) is completely conserved in every prokaryote genome according to previous reports [2]. Recent accelerated accumulation of complete genomic sequences motivated us to conduct an updated screening for anti-SD sequence variants.

## Results

**Figure 1:**
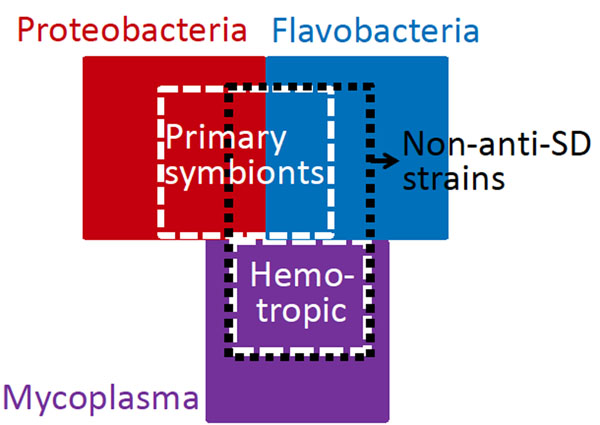
Distribution of the non-anti-SD genomes

By comparison analysis of 1,182 completely sequenced eubacterial genomes, we found fifteen genomes in which no 16S rRNA genes carry the anti-SD motif. Loss of the anti-SD motif in these ‘non-anti-SD’ genomes is always accompanied by loss of SD-like sequences in 5’ UTR. These non-anti-SD genomes belong to either the phylum Proteobacteria (n=3), the class Flavobacteria (n=9), or the genus Mycoplasma (n=3) (Figure 1). Bacteria with extreme association with eukaryotic hosts, such as primary symbionts of insects (n=9) and intracellular parasites (n=3), were prevalent (12 of 15) in the non-anti-SD genomes. Their loss of SD-related features may be related to the massive gene/function decay driven by the host association. All Flavobacteria strains we examined, regardless of anti-SD motif conservation, seem to rarely depend on SD/anti-SD interaction to initiate translation because significant A enrichment in 5’ UTR of their genes will not allow the C-rich anti-SD sequence to bind. An unknown alternative mechanism that uses the A-rich sequence may play a dominant role for translation initiation in this lineage, resulting in many non-anti-SD genomes that even include free-living bacteria’s. In Mycoplasma, only those living in red blood cells (hemotropic mycoplasmas) have lost the anti-SD motif (Figure 1), suggesting that certain environmental factors may have been involved in this loss.

## Conclusions

The non-anti-SD genomes we reported here represent an extreme aspect of the dynamic evolution in the translation initiation mechanisms, strongly indicating extinction of SD/anti-SD interaction in some bacterial lineages. Attentive analysis of such peculiar genomes led us to suggest likely factors that gave rise to the loss. First, advanced association with eukaryotic hosts and subsequent immense genomic/functional reduction. Second, intra-genomic dominance of SD-independent translation initiation mechanisms. Finally, hemotropic environment.

Our study described here has been published as an original research article [3].
